# Development and validation of the interactive health literacy scale for college students majoring in kinesiology

**DOI:** 10.3389/fpsyg.2025.1647504

**Published:** 2026-01-06

**Authors:** Miaomiao Wen, Xiaorui Wang, Zhihua Yin

**Affiliations:** 1 School of Physical Education and Sports, Central China Normal University, Wuhan, China; 2 Department of Social Sports, Hebei Sport University, Shijiazhuang, China; 3 College of Physical Education and Health, East China Normal University, Shanghai, China

**Keywords:** interactive health literacy, college students majoring in kinesiology, scale development, exploratory factor analysis, confirmatory factor analysis

## Abstract

**Introduction:**

Interactive health literacy is crucial for sports major college students. However, previous studies have focused on qualitative research, resulting in a lack of quantitative research and a lack of samples from Eastern countries. This study aimed to develop and validate the Interactive Health Literacy Scale for College Students Majoring in Kinesiology (IHLS-CSMK).

**Methods:**

A questionnaire was created based on the 60 items of the structural model described in our previous research. Using a questionnaire survey, quantitative data was collected from a sample of 964 sports major college students. We used SPSS 27.0 and AMOS 24.0 to analyze the data and conducted project analysis, exploratory factor analysis, and confirmatory factor analysis to test the data. The IHLS-CSMK consists of 28 concepts, namely mastering health information, promoting health interaction, and solving health problems. The results indicate that IHLS-CSMK has good content validity, internal construct validity, and internal consistency.

**Results and discussion:**

Chinese college students majoring in kinesiology have similarities and differences in interactive health literacy compared to students from other nations. Suggestions to improve the interactive health literacy of college students majoring in kinesiology include: (1) optimizing health education courses and training; (2) enhancing digital and technological support; (3) strengthening diversified health literacy assessment and feedback; and (4) supporting campus environments. The IHLS-CSMK has achieved significant results in China; however, due to differences in cultural backgrounds, health concepts, and education systems, further validation is needed to determine whether this scale is applicable to other countries.

## 1 Introduction

Health literacy has gained attention worldwide in recent years as a new field of study. It is used both as an objective for educating and promoting health and as a means of evaluating the outcomes of interventions (U.S. Department of Health and Human Services, 2000). Health literacy typically covers three major areas: healthcare, disease prevention, and health promotion. Internationally recognized is Nutbeam D’s tripartite approach, which divides health literacy into functional health literacy, interactive health literacy, and critical health literacy. Functional health literacy refers to an individual’s ability to read and calculate health-related information, which is the most fundamental ability; Interactive health literacy is related to higher-level health information cognitive literacy and social skills, used to obtain and apply information from different communication media to cope with changing environments; Critical health literacy is the ability of individuals to critically analyze information and influence larger life events and contexts through individual and collective actions. Interactive health literacy is a high-level health literacy, which can be used to evaluate an individual’s ability to acquire, understand, and apply health information, thereby improving patients’ understanding, satisfaction, and willingness to adhere to healthy treatment plans (Duggan, 2006; Frosch and Elwyn, 2014; Koh et al., 2013). Interactive health literacy focuses on developing individual skills, such as problem solving, communicating, and making healthy choices, and the ability to process health knowledge (Nutbeam, 2009; Canadian COL, 2008). Health education activities in public places such as schools and neighborhoods are essential to improve interactive health literacy (Mark et al., 2006; De Walt et al., 2004). Health literacy has different definitions and conceptual models from the perspectives of public health and clinical medicine (Pleasant AKuruvilla, 2008; Nutbeam, 2008; WHO, 1998); however, both sectors agree that health education holds an important role in clinical and community health literacy (Coulter and Ellins, 2007). Interactive health literacy emphasizes that individuals should not only have the ability to access and understand health information but also be able to effectively apply this information to solve practical health problems. This includes the ability to acquire, understand, apply, and disseminate health information, which is an important literacy that sports majors must possess in their academic and professional careers. Improved interactive health literacy can help college students manage their own health, cultivate health decision-making abilities, and disseminate health information to others through physical education. Interactive health literacy emphasizes the role of health education, especially in enhancing individuals’ self-efficacy and health management abilities. College students majoring in sports hold an important role as health communicators in their daily studies and future careers. Therefore, improving their interactive health literacy not only aids their personal health management, but also promotes the wider dissemination of health education and advances the population’s overall health literacy.

Individual health outcomes are affected by lower levels of health literacy, which are prevalent worldwide (Adams et al., 2009; Canadian COL, 2008; De Walt et al., 2004). Those with low levels of health literacy have little access to health education (Williams et al., 2002), are ill-equipped to prevent chronic disease, and are 1.5 to 3 times more likely to have a poor health outcome (Sun and Fu, 2011; Nutbeam, 2000). Health literacy is reported to be highly correlated with health education and empowerment; the means of health education enhances literacy and self-efficacy, which in turn improves interactive thinking (Coulter and Ellins, 2007). Health education and health promotion are widely recognized internationally as a means of improving a population’s health literacy level, which is an indicator of the effectiveness of health education and interventions (Nutbeam, 2000).

College students with increased health literacy can lead to an improved health industry and thus promote people’s awareness of “active health” (Zhihua et al., 2018). Some scholars have found that college students’ majors affect their health literacy and that health education at the college level can improve college students’ health literacy. It is easier to improve college students’ health literacy when they are exposed to health and medicine related majors during their college years (Rababah et al., 2019; Mullan et al., 2017). As a special group with long-term exposure to sports, college students majoring in kinesiology are knowledgeable of sports activities, which can promote health literacy. The health behaviors and habits that they develop in school, the health knowledge that they reserve, and the work that they engage in after graduation are closely linked to improving public health literacy. College students majoring in kinesiology usually have a certain understanding and awareness of health and sometimes have a high level of health literacy. They are not only receiving health knowledge but are becoming important disseminators of health knowledge. College students majoring in kinesiology usually have strong physical fitness and athletic ability, but a gap remains in the acquisition and application of health information. Compared to ordinary college students, they are exposed to more content about health and exercise in their professional learning, but the interactive component of health literacy, such as the ability to disseminate and communicate health information, is often overlooked. Therefore, to enhance the interactive health literacy of this group, it is not only necessary to focus on their learning of health knowledge, but also to strengthen their ability to apply this knowledge in daily life and share health information with others.

A considerable amount of research has explored the perceptions of improving health literacy among college students; however, little attention has been paid to interactive health literacy among specific groups of kinesiology students in Eastern countries. To fill this gap, Wen et al. (2025) have conducted a study in China with many kinesiology students. Although their research utilized qualitative research methodology with a bottom-up rooted theory base, they comprehensively investigated the structure of interactive health literacy among university students in the field of kinesiology and identified the essential elements of their interactive health literacy. Qualitative research is inherently more subjective than quantitative research and usually relies on fewer participants. Some studies have used quantitative methods through questionnaires to investigate health literacy levels among college students (Rababah et al., 2019; Mullan et al., 2017; S-S Budhathoki et al., 2019). Of note, the examination of healthy literacy among college students majoring in kinesiology in eastern countries remains under study. Therefore, further objective quantitative research is needed to validate and refine a scientific set of scales to assess the interactive health literacy of kinesiology students. A reliable and practical means of universally assessing the level of interactive health literacy among kinesiology students is needed. In addition, the development and implementation of such a tool may improve the understanding of, and address, current methods of health literacy among college students.

Currently, 32 health literacy assessment scales, which are used to measure a subject’s ability to understand and apply health knowledge in a specific context, have been developed; 21 of them can assess interactive health literacy (Weiss et al., 2005; S-S Budhathoki et al., 2019; Baron-Epel et al., 2007). The Measure of Adolescent Health Literacy (MAHL) (Massey et al., 2013) is a multidimensional scale specifically for adolescents and includes doctor-patient communication, interpersonal interaction, and the use of health information. The Brief Health Literacy Screen (BHLS) (Wallace et al., 2006) is the most concise scale among the tools for measuring interactive health literacy, which quickly evaluates the patient’s ability to utilize healthcare services through only three entries. To address the lack of a validated tool for measuring the level of interactive literacy among kinesiology students, we created a structural model of interactive health literacy (Wen et al., 2025) and administered the questionnaire to 964 college students majoring in kinesiology in China to assess the reliability and validity of the scale. The IHLS-CSMK developed and described here has the potential to be a valid and reliable tool. Additionally, it may highlight approaches to improve interactive health literacy for university students majoring in kinesiology.

With the global emphasis on health, improving health literacy has also attracted more attention. College students are the main force in the development of social health, and improving their interactive health literacy can directly bring new health experiences to more people. This research developed and validated the Scale for Measuring Interactive Health Literacy for College Students Majoring in Kinesiology (IHLS-CSMK). This study will expand the relevant research on health literacy assessment scales at home and abroad. Based on the impact of different majors on the health literacy of college students, it can discover the promoting effect of sports majors on health literacy. This will contribute to an international health literacy assessment scale. Therefore, this study has rich theoretical and practical value.

## 2 Methods

Written informed consent was obtained from all participants for the publication of potentially identifiable images or data included. The research team maintained the confidentiality of participant information. All interviewees signed the interview opinion request form and agreed to participate before the interview. The interview process was recorded and participants’ privacy was guaranteed.

### 2.1 Item generation

This study used a convergence model (Creswell and Clark, 2017) to analyze the qualitative and quantitative data. By triangulating the findings, a more complete understanding of the issues emerged. Data was collected during the qualitative phase of the study, as previously published (Wen et al., 2025)^.^ The quantitative phase validated the findings and provided insight into the perceptions of interactive health literacy among college students majoring in kinesiology,as below described.

Wen et al. (2025) conducted a comprehensive review of existing literature using a mixed research approach. Firstly, a comprehensive literature search was conducted on the structure model of interactive health literacy among kinesiology major college students using domestic and foreign databases. A total of 36 Chinese articles and 195 English articles were collected, and preliminary concepts and theoretical frameworks were analyzed and proposed. Secondly, an interview outline was compiled to conduct semi-structured interviews with 17 kinesiology major college students, kinesiology teachers, and kinesiology health experts, in order to gain a deeper understanding of their views and experiences on using the advantages of sports majors to enhance interactive health literacy. Finally, the collected textual data will be organized and summarized, and Nvivo14 software will be used for coding analysis. The grounded theory research method will be used to derive a structural model of interactive health literacy among kinesiology major college students. This model consists of 3 main categories: Harnessing Health Information, Promoting Health Interactions, and Addressing Health Problems, 8 subcategories, and 60 concepts. These 60 concepts can provide a comprehensive description of the structural model, therefore, they were used in the Pretest version of IHLS-CSMK construction, and detailed information can be found in Supplementary Table S1. Before conducting the survey, the researchers distributed the questionnaire to 2 experts and 10 students, and conducted preliminary research on the completion time of the questionnaire. The results showed that the completion time of the questionnaire in this study was controlled within 2–3 min, which could reduce the risk of response fatigue and superficial answers for the investigators.

### 2.2 Participants and procedure

A pre-test version of the IHLS-CSMK was developed and consisted of 60 items on health literacy and six items on basic details (sex, ethnic, geographic, year, university type and level). The IHLS-CSMK is scored on a five-point Likert scale (1 = strongly disagree, 2 = disagree, 3 = undecided, 4 = agree, and 5 = strongly agree), which is commonly used in health literacy measurement studies. Higher scores indicated higher levels of interactive health literacy among kinesiology students, or the increased importance of an influential factor. The IHLS-CSMK was carried out through sojump1, a professional online survey platform that is widely used in China (Zhang et al., 2017; Mei and Brown, 2018). In order to reduce the risk of robot answering and shallow answering, 5 reverse questions were set in the survey questionnaire of this study. The geographical distribution of respondents can be tracked according to their IP addresses and real answers. Due to the limitations of the platform, the reliability of the data may be weakened, which is also one of the shortcomings of this study. In future survey research, we will strengthen the control of collected data. To ensure the reliability of the returned IHLS-CSMK, the system recorded the time taken by respondents to submit and complete the survey, IP addresses, and cities.

This study distributed a survey questionnaire to 964 college students major in kinesiology and analyzed the collected data. For testing internal structural validity of the IHLS-CSMK, we used three different samples, following the recommendations of Costello and Osborne (Costello and Osborne, 2005): 123 questionnaires collected from the pre-test version of IHLS-CSMK were used for item analysis; 429 questionnaires collected from the beta version of IHLS-CSMK were used for exploratory factor analysis (EFA); and 412 questionnaires collected from the final version of IHLS-CSMK were used for validation factor analysis (CFA).

First, from October 2023 to November 2023, we administered the pre-test version of the IHLS-CSMK to 123 Chinese college students majoring in kinesiology. Further details of this sample are provided in Supplementary Table S2.

Next, based on the data analysis of the pre-test version, a beta version of the IHLS-CSMK was created and distributed to 429 Chinese kinesiology students, as shown in Supplementary Table S3. Different samples were collected for EFA. Participants were recruited using the same procedures described above, and samples were collected at two different times using different internet links between December 2023 and January 2024.

Finally, we created the final version of the IHLS-CSMK, which consists of 28 programs. We distributed the final version of the IHLS-CSMK to 412 Chinese college students majoring in kinesiology. The samples were collected for CFA. Participants were recruited using the procedures described above and samples were collected at different times using different internet links between February 2024 to March 2024. For more details on this sample, see Supplementary Table S4.

### 2.3 Measures and statistical Analyzes

First, we conducted item analyses on the first sample (*n* = 123) to assess the appropriateness, feasibility, and relevance of the items. We used the critical ratio method to identify items for deletion based on the attainment of critical values and performed homogeneity and correlation analyses. As the IHLS-CSMK is scored using a five-point Likert scale, it is common practice to use the critical ratio method and correlational analysis to analyze items (Bryant and Yarnold, 1995).

Second, for statistical determination of the number of factors and items to be retained in the IHLS-CSMK, we conducted a series of EFAs on the second sample (*n* = 429). We used Bartlett’s test of Kaiser-Meyer-Olkin (KMO) values and variable rotation, which is the method recommended by Kaiser (Kaiser, 1974) for scale development. Following Tabachnick and Fidell (Tabachnick and Fidell, 2001), KMO tests with KMO values of 0.60 and higher are essential for good factor analysis. To determine the number of factors, we then performed factor analysis (Matsunaga, 2010; Wu, 2010; Escobar-Viera et al., 2018). The criteria were as follows: (1) each item had > 0.40 commonality; (2) each factor contained at least three items; and (3) the items were consistent with the meaning of the factor and difficult to combine with other items.

Finally, EFA provides a more rigorous approach for checking structural validity by comparing different *a priori* theoretical models (Bryant and Yarnold, 1995). Prior to the EFA, we assessed of the reliability and validity of the final version of the IHLS-CSMK using a third sample (*n* = 412). Three-factor and structural equation models were tested for the IHLS-CSMK, as derived from the EFA and CFA, using AMOS version 24.0. To determine an acceptable model fit, we evaluated the following criteria: GFI, IFI, NFI, TLI, CFI > 0.90, and RMSEA < 0.1 (Hu and Bentler, 1999; Fan, 1954).

## 3 Results

### 3.1 Item analysis

A t-test for independent samples was used to investigate the relationship between the IHLS-CSMK items and the total IHLS-CSMK score by dividing them into high (27% before the total score) and low (27% after the total score) subgroups (Ebel, 1951). Based on the criterion of significant differences, some items were excluded; 25 items were not in line with criteria, and the other items were significantly discriminated against (t-value = 2.949 < 3). See Supplementary Table S1 for details. These results indicate that the rest of the entries in the forecast version of the IHLS-CSMK had a high level of discrimination and a high level of discriminatory power.

Correlation analysis was used to explore the relationship between the total score and the individual scores of the IHLS-CSMK. Precisely guided by correlation coefficient criteria, items with correlating coefficients that did not meet the criteria were eliminated. This includes Kaiser’s (1960) developmental recommendations.

Items with a differentiation index less than 0.20 were eliminated from the scale. To assess the relationship between each item and the total score of the IHLS-CSMK, Pearson’s correlation analyses were conducted for each of the 123 returned pre-test versions of the IHLS-CSMK. The correlations between the 60 individual items and the overall IHLS-CSMK score were highly significant (*p* < 0.01, see Supplementary Table S5), correlating with each other to exceed 0.40. This indicated that each item associated with the overall IHLS-CSMK score was highly correlated and closely related to psychological traits or underlying behavior.

Based on the results of the critical ratio and correlation analysis methods, items 21 and 25 were removed from the IHLS-CSMK. As a result, 58 items were considered suitable and retained for continued development and validation of the IHLS-CSMK.

### 3.2 Exploratory factor analysis

In Wen et al.’s study, it was found that the interactive health literacy structure model of sports major college students includes three main dimensions: Harnessing Health Information, Promoting Health Interactions, and Addressing Health Problems. This study used the three dimensions in the structural model as the three factors for exploratory factor analysis. The IHLS-CSMK questionnaire (beta version) was created for EFA, which contained the remaining 58 items. The KMO and Bartlett tests, maximum variance tests, and factor naming were performed during EFA.

#### 3.2.1 KMO and Bartlett’s tests

To assess whether the IHLS-CSMK applies to EFA, the KMO and Bartlett tests were performed on 58 items using the IHLS-CSMK (beta). Results for Bartlett’s spherical test presented a KMO value of 0.976 and a *χ*^2^ value of 5959.5, which were highly statistically significant (*p* < 0.01).

The KMO value was 0.976, indicating no significant difference in the correlation between the items and thus deeming it suitable for factor analysis. Additionally, the original hypothesis was rejected by the results of Bartlett’s sphericity test, indicating that the items of the IHLS-CSMK were not independent; common factors were found between the correlation matrices of the pro groups, rendering them suitable for factor analysis. These results provided consistent information supporting the suitability of the IHLS-CSMK for EFA (Table 1).

**Table 1:** Variance explained results for the IHLS-CSMK (Beta version) (*n* = 429).

Factor number	Characteristic roots			Explanation of variance before rotation			Explanation of variance after rotation		
	Characteristic roots	Explanation of variance %	Cumulative %	Characteristic roots	Explanation of variance %	Cumulative %	Characteristic roots	Explanation of variance %	Cumulative %
1	13.853	49.476	49.476	13.853	49.476	49.476	6.815	24.34	24.34
2	1.762	6.292	55.768	1.762	6.292	55.768	6.229	22.246	46.586
3	1.037	3.705	59.473	1.037	3.705	59.473	3.608	12.887	59.473
4	0.765	2.733	62.206						
5	0.755	2.698	64.903						
6	0.72	2.573	67.476						
7	0.64	2.287	69.763						
8	0.629	2.246	72.01						
9	0.556	1.985	73.995						
10	0.521	1.862	75.857						
11	0.501	1.79	77.647						
12	0.497	1.774	79.422						
13	0.463	1.655	81.077						
14	0.448	1.599	82.676						
15	0.442	1.579	84.255						
16	0.423	1.511	85.767						
17	0.418	1.494	87.261						
18	0.395	1.411	88.672						
19	0.379	1.353	90.025						
20	0.368	1.313	91.338						
21	0.364	1.302	92.64						
22	0.348	1.241	93.881						
23	0.327	1.167	95.049						
24	0.31	1.108	96.157						
25	0.287	1.025	97.182						
26	0.281	1.002	98.184						
27	0.264	0.944	99.128						
28	0.244	0.872	100						

#### 3.2.2 Maximum variance

After conducting text analysis on IHLS-CSMK (Beta version), it was found that deleting items resulted in lengthy and repetitive statements (such as 7 Better understanding of health information in the context of one’s own experience), unrealistic statements (such as 14 Be able to ensure the accuracy of the health information disseminated), and redundant concepts (such as 32 Solving health problems requires knowledge of health status).

According to the varimax rotated principal component analysis of the first 58 items of the IHLS-CSMK (beta), 28 items were retained in accordance with the Kaiser-Guttman rule (Cronbach, 1951).

Items 2, 3, 4, 7, 8, 9, 11, 14, 19, 27, 28, 30, 31, 32, 33, 34, 35, 36, 37, 39, 40, 41, 42, 43, 44, 45, 51, 56, 57, and 58 were deleted based on a loading factor threshold of < 0.4. The remaining 28 items were compliant (see Supplementary Table S6). The loadings of the factors ranged from 0.610 to 0.725, with a commonality of 0.40 across the IHLS-CSMK entries. Factor 1 (12 items) included items 15, 16, 17, 18, 20, 21, 22, 23, 24, 25, 26, and 29. Factor 2 (10 items) included items 38, 46, 47, 48, 49, 50, 52, 53, 54 and 55. Factor 3 (6 items) included items 1, 5, 6, 10, 12, and 13.

**Figure 1:**
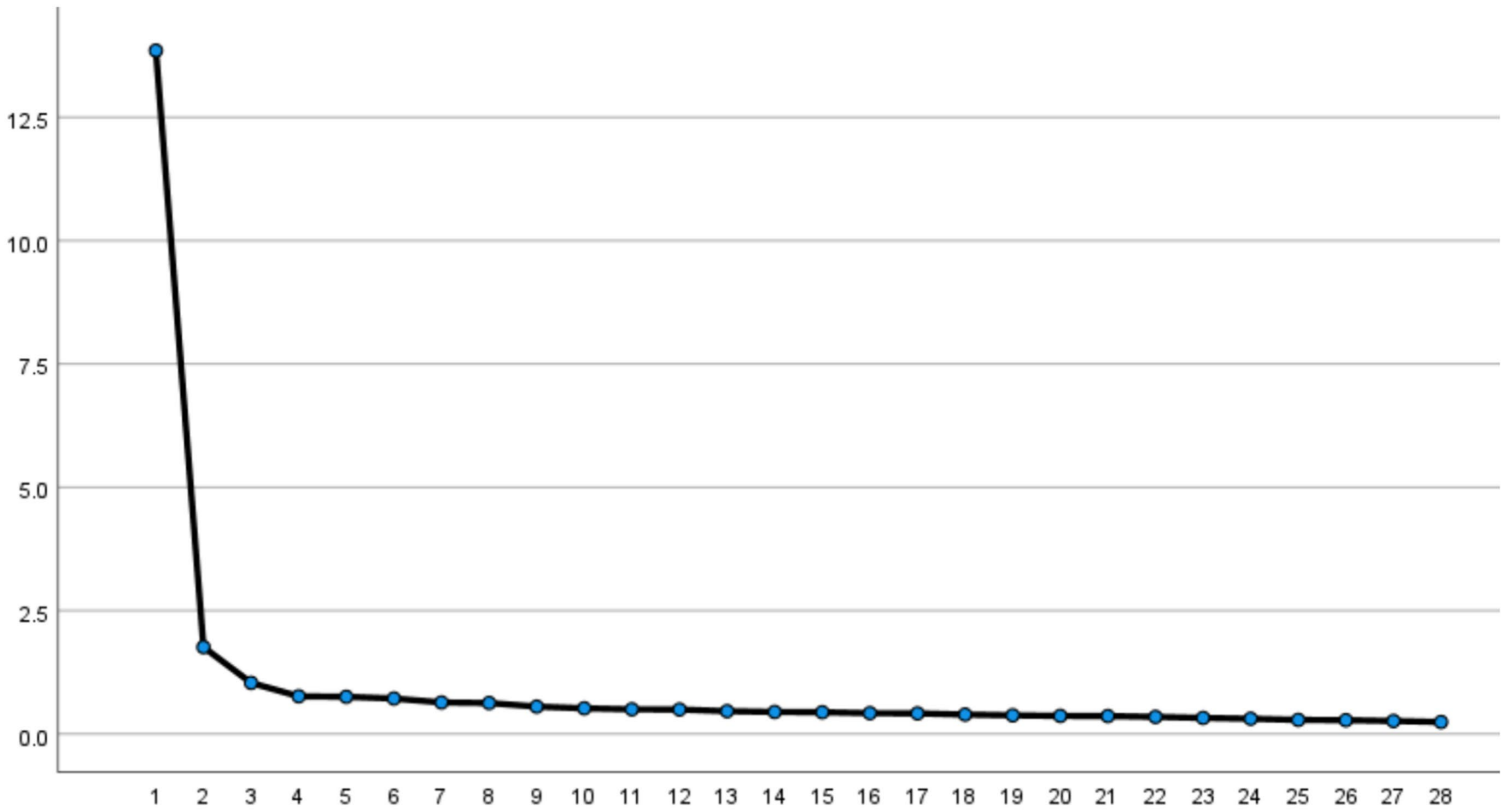
Gravel map.

In addition, the steep slope plot (Figure 1) shows that the slope flattens after the third principal component, indicating that it is appropriate to retain all three factors.

#### 3.2.3 Factor naming

Analysis of the IHLS-CSMK items indicated that all items meet professional expectations of the health literacy field. The factors and items were highly consistent; we named Factor 1 “Promoting Healthy Interactions,” Factor 2 “Solving Health Problems,” and Factor 3 “Having Health Information.” After completing the EFA, we deleted items 2, 3, 4, 7, 8, 9, 11, 14, 19, 27, 28, 30, 31, 32, 33, 34, 35, 36, 37, 39, 40, 41, 42, 43, 44, 45, 51, 56, 57, and 58. Thirty items were deleted from the projected version of the IHLS-CSMK (Table 2).

**Table 2:** Rotation matrix of three factor loadings of 30 items in the IHLS-CSMK (Beta version) (*n* = 429).

Items	Public factor		
	Factor 1	Factor 2	Factor 3
A23. More warning cases can arouse college students’ attention to health.	0.725	0.229	0.249
A18. Visualization of external devices enables monitoring of one’s own well-being.	0.71	0.261	0.21
A22. More health promotion can make college students pay more attention to health.	0.689	0.372	0.135
A29. Physical activity enhances physical fitness and related motor skills.	0.673	0.418	0.138
A25. Enhancement of health awareness through learning health courses.	0.666	0.432	0.171
A26. Schools can provide more health interventions to promote health awareness.	0.665	0.36	0.236
A16. Mental health problems can affect physical health.	0.656	0.363	0.229
A21. A good state of health can address the psychological stress of training or studying.	0.645	0.355	0.189
A15. Physical health is the basic state of life.	0.637	0.329	0.296
A20. Regular physical activity can regulate mental health status.	0.631	0.395	0.213
A24. Enhancing the knowledge of prevention of common diseases and medication can show health awareness.	0.619	0.444	0.224
A17. Solving health problems requires knowledge of health status.	0.61	0.435	0.184
A52. Healthy lifestyle habits and exercise behaviors require strong self-discipline.	0.365	0.697	0.213
A53. Pre-exercise warm-up and post-exercise stretching are effective in preventing sports injuries.	0.409	0.689	0.193
A46. Physical exercise can make the body in better shape.	0.409	0.683	0.092
A49. Maintaining healthy habits can help make good health decisions.	0.359	0.657	0.233
A50. Healthy living requires behavioral habits such as eating, resting and exercising.	0.396	0.649	0.23
A48. Preventing sports injuries through good preparation for exercise.	0.343	0.647	0.24
A54. Be able to acquire knowledge of health-promoting exercises.	0.321	0.644	0.278
A55. Regular participation in physical activities enhances social interaction skills.	0.386	0.64	0.26
A38. Engaging in regular physical activity to maintain one’s health status.	0.344	0.639	0.264
A47. Exercise experience can help determine health problems such as sports injuries.	0.333	0.628	0.208
A12. Be able to perform standard exercise movements that promote physical fitness.	0.249	0.079	0.701
A1. Being able to get more health information from schools.	0.045	0.158	0.664
A10. Be able to consult teachers or professionals to help them understand health information.	0.196	0.147	0.656
A5. The examination can strengthen the mastery of health information and improve health literacy.	0.111	0.356	0.64
A13. Exercise experiences can help to better understand related chronic exercise injuries and diseases.	0.198	0.17	0.633
A6. Being able to access health information from the WeChat public platform.	0.24	0.191	0.622

### 3.3 Scale structure exploration based on confirmatory factor analysis

Reliability and validity of data from the third round of the IHLS-CSMK (*n* = 412) were analyzed prior to the CFA exploring the structure of the IHLS-CSMK.

#### 3.3.1 Reliability analysis

The internal consistency of the IHLS-CSMK was tested using Cronbach’s coefficient alpha. As shown in Supplementary Table S7, the overall alpha coefficient of the IHLS-CSMK was 0.962. The alpha coefficients of the factors mastering health information, facilitating health interactions, and solving health problems were 0.794, 0.943, and 0.932, respectively. The above results indicate that the consistency or homogeneity of the items on the IHLS-CSMK scale is good. This means that the scale is highly reliable.

#### 3.3.2 Folded half reliability

The remaining 28 items were divided into two parts after eliminating the required items. The results are shown in Table 3. Cronbach’s alpha coefficient value for the first half was 0.908, and the alpha coefficient value for the second half was 0.948; both were above 0.8. In addition, the first and second half correlation coefficient was 0.867, and the collapsed confidence coefficient was 0.918; both of which were greater than 0.8, suggesting a high level of confidence. According to Cronbach’s (Cronbach and Meehl, 1955) criterion, half-time reliability greater than 0.8 indicates high-quality reliability (Tucker, 1949). Therefore, all scales were in accordance with the criteria and were included in the subsequent analyses. Further examination of the consistency of individual items across the IHLS-CSMK dimensions was conducted; Table 3 provides statistical information on the corresponding items of the three dimensions.

**Table 3:** Results of discounted half confidence analysis (n = 429).

Discount factor		Guttman coefficient	Cronbach’s alpha coefficient				Correlation coefficient values
Equal length	Unequal length	0.918	First half		Second half		0.867
			Value	Number of items	Value	Number of items	
0.929	0.929		0.908	14	0.948	14	

According to psychometric prerequisites, Cronbach’s alpha coefficients above 0.9 show good reliability, and a dichotomous reliability greater than 0.8 indicates high reliability. Based on these criteria, the scale was found to comply with standards and was used to continue analysis. The consistency of each of the internal dimensions of the IHLS-CSMK was further examined; Table 4 provides statistics on the total number of corresponding items of the three dimensions.

**Table 4:** Overall statistics for IHLS-CSMK items for each dimension (*n* = 429).

Dimensionality	Title item	Dimensions when items are deleted average	When an item is deleted the scale variance of	Amended total project relevance	Relevance Square	Cronbach at the time of item deletion alpha factor
Harnessing health information	A1	111.91	335.906	0.373	0.291	0.962
	A5	112.27	329.913	0.530	0.424	0.961
	A6	112.28	330.264	0.503	0.386	0.962
	A10	112.16	332.085	0.461	0.347	0.962
	A12	112.19	331.493	0.471	0.395	0.962
	A13	112.08	333.638	0.468	0.36	0.962
**Promoting health interactions**	A15	112.34	320.182	0.724	0.576	0.960
	A16	112.34	317.85	0.73	0.599	0.960
	A17	112.29	320.158	0.727	0.579	0.960
	A18	112.36	320.777	0.693	0.554	0.960
	A20	112.25	319.963	0.728	0.567	0.960
	A21	112.24	322.478	0.701	0.543	0.960
	A22	112.3	320.104	0.719	0.583	0.960
	A23	112.34	320.692	0.700	0.565	0.960
	A24	112.28	318.878	0.756	0.612	0.960
	A25	112.31	318.034	0.758	0.637	0.960
	A26	112.33	319.072	0.738	0.611	0.960
	A29	112.36	318.829	0.739	0.606	0.960
**Addressing Health Problems**	A38	112.29	320.951	0.711	0.563	0.960
	A46	112.28	320.875	0.711	0.583	0.960
	A47	112.28	323.219	0.673	0.539	0.960
	A48	112.34	320.831	0.706	0.548	0.960
	A49	112.25	320.913	0.720	0.558	0.960
	A50	112.3	319.333	0.739	0.594	0.960
	A52	112.24	320.622	0.741	0.638	0.960
	A53	112.24	317.797	0.758	0.638	0.960
	A54	112.26	321.52	0.704	0.559	0.960
	A55	112.21	321.243	0.740	0.587	0.960

#### 3.3.3 Validity analysis

Several types of scale validity tests were carried out, including structural, content, and calibration validity. However, as the IHLS-CSMK remains in its early stages, comparable scales are lacking both nationally and internationally. Therefore, content and structural validity tests were this study’s focus.

#### 3.3.4 Content validity

A systematic process was used to develop the scale, which included an extensive literature review, online post-collection, personal interviews, and ongoing expert consultations (Wen et al., 2025). First, a domestic and international literature review of interactive health literacy among college kinesiology students was conducted, and 36 articles in Chinese and 195 articles in English were collected and analyzed to extract preliminary concepts that supported the study’s theoretical framework. Second, semi-structured interviews were conducted with 17 college kinesiology students, physical education teachers, and physical fitness experts, who were selected via a rigorous coding process based on grounded theory. The concepts derived from this systematized theory were used as the basis for developing the project. Finally, five professors of physical education oversaw the project, and with the joint efforts of experts and research assistants, the scale was designed to be modified and adjusted through feedback from several rounds of testing. Consequently, the process of developing the scale met the criteria for content validity testing and was scientifically valid.

#### 3.3.5 Structural validity

Structural validity testing is often used to verify the correlation between the theoretical structure of hypotheses and scales, among which factor correlation analysis and confirmatory factor analysis are commonly used analytical methods. The structural validity test in this study is mainly used to verify the correlation between the overall scale developed and its inherent dimensions of “Harnessing Health Information, Promoting Health Interactions, and Addressing Health Problems.” According to Tucker’s (1949) theory, a reasonable level of construct validity refers to the correlation between items and the total score of the scale being between 0.30 and 0.80, and the standard between factors being between 0.10 and 0.60, which is generally accepted as good construct validity (Creswell and Clark, 2017). Ensuring that the correlation index is within a reasonable standard range is sufficient. If the correlation index is too high, it indicates that there is an overlapping relationship between the dimensions; If the relevant index is too low, it indicates that there is a significant deviation between each dimension and the overall scale.

The correlations between the three dimensions and the total IHLS-CSMK score were 0.715, 0.955, and 0.947, respectively,the correlation coefficients all meet the criteria for structural validity testing, indicating that the existence of the three dimensions of the evaluation scale has good independence and good correlation with the overall scale, indicating that the scale has good structural validity. Thus, a degree of independence between the factors within the IHLS-CSMK was indicated by the observed correlation coefficients and each IHLS-CSMK factor measured its intended target. Thus, the IHLS-CSMK showed good construct validity (Supplementary Table S6).

#### 3.3.6 Confirmatory factor analysis

Confirmatory factor analysis is used to evaluate the rationality and feasibility of the constructed structural model. The main purpose of this study is to verify the fit between the interactive health literacy structural model of kinesiology major college students constructed by Wen et al. using grounded theory and the collected data, and to examine the structural validity of the scale. In this section, AMOS software will be used to establish a structural equation model, aiming to verify the scientific validity and model fitting of the theoretical model.

The following criteria should be considered for the construction of an ideal theoretical model: (1) the range of the GFI, NFI, TLI, CFI, and IFI should be between 0 and 1, where values closer to 1 indicate an improved fit; (2) lower *χ*^2^ values with *χ*^2^/df ≤ 5 (if 1 < *χ*^2^/df < 2, the fit is considered better); and (3) lower RMSEA values with RMSEA ≤ 0.05 (RMSEA ≤ 0.08 is also acceptable). In addition, when *χ*^2^/df < 2, RMSEA < 0.05, and the values of GFI, AGFI, NFI, TLI, CFI and IFI are greater than 0.90 (higher than 0.80 is also acceptable), the model is considered well fitted.

**Figure 2:**
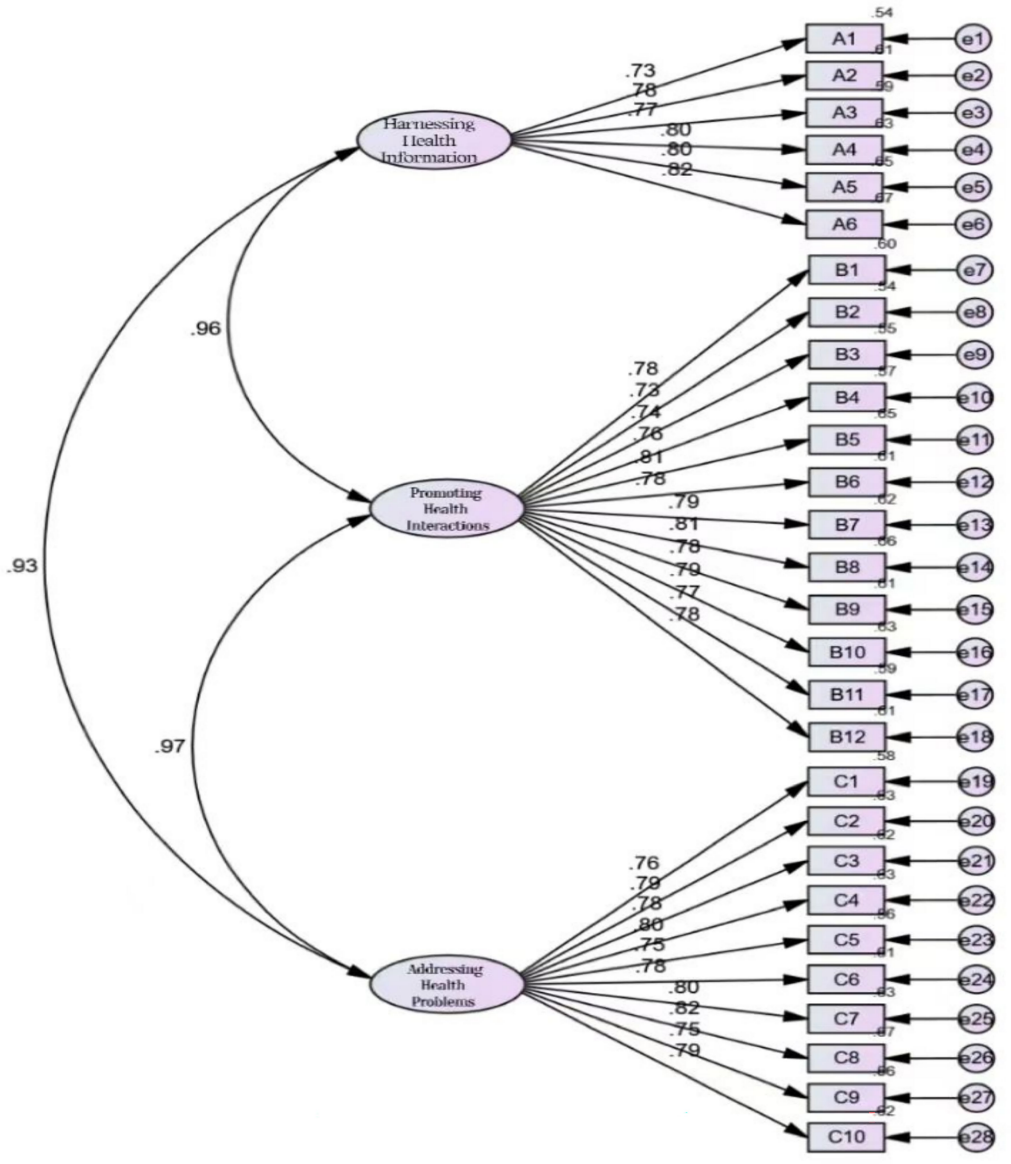
A three-factor model of the IHLS-CSMK.

This study used AMOS software to conduct structural equation modeling analysis on 412 valid answer sheet data collected. The IHLS-CSMK developed through exploratory factor analysis contained 3 factors. When constructing the structural equation model, confirmatory factor analysis was conducted using these 3 observed variables to verify the scientific validity of the theoretical model. To accomplish this, a covariance matrix was obtained for the sample and used for model validation. The result is:*χ*^2^/df = 1.773, RMSEA = 0.043, GFI = 0.907, CFI = 0.971, IFI = 0.971, TLI = 0.968, and NFI = 0.935,indicate that the overall theoretical model of the scale is well fitted. The results obtained in this study are not significantly different from the Short Assessment of Health Literacy—Spanish and English (SAHL-S&E), which has been developed and the result was *χ*^2^/df = 1.35, TLI = 0.989, RMSEA = 0.042 (Lee et al., 2010). This is evidence of standard validity. These results all meet the standards of ideal theoretical model fitting indicators, indicating that the three factors in the interactive health literacy of kinesiology major college students determined through exploratory factor analysis have been validated by formal quantitative research data, demonstrating the rationality and effectiveness of this structure. The three-factor structural model is shown in Figure 2.

## 4 Discussion

This research developed a rigorous psychometric scale to create the IHLS-CSMK, which consisted of 28 items across three dimensions: mastering health information, facilitating health interactions, and solving health problems. There were six items in the health information mastery dimension, 12 items in the health interaction facilitation dimension, and 10 items in the health problem-solving dimension. The focus of our previous publications was on the qualitative phase of the study, generating a wealth of analyzed data. In our study, we present the results of the quantitative phase, which are a validation of previous findings and a source of new insights into the structure of interactive health literacy among college kinesiology students. Specifically, Wen et al.’s (Wen et al., 2025) construction model revealed the existence of three dimensions, which means that our study is consistent with previous research and theories.

The main purpose of analysis was to assess the differences among the IHLS-CSMK programs to avoid redundancy and ensure that each focused on different items. For accuracy assurance, critical ratios and correlation analysis were used to cross-validate. In detail, independent samples t-tests were performed on the 27% high and 27% low subgroups using the critical ratio method. Having differentiated and correlated, items 21 and 25 did not meet the criteria, the remaining 58 tests were highly discriminatory (*p* < 0.01). This indicates good discriminatory power. Correlation analysis showed that the correlation coefficients between each IHLS-CSMK item and the total IHLS-CSMK score were higher than 0.40, indicating high uniformity. The combination of the results of the critical ratio method and the correlation analysis, the items of the predictive version of the IHLS-CSMK were found to be highly independent but highly correlated with the total IHLS-CSMK score, confirming the appropriateness of the items. An EFA was then performed on the IHLS-CSMK (beta version), which contained 58 items based on the 429 questionnaires received. Twenty-eight components were obtained after applying forward cross-rotation with maximum variance principal component analysis (varimax). However, the 2nd, 3rd, 4th, 7th, 8th, 9th, 11th, 14th, 19th, 27th, 28th, 30th, 31st, 32nd, 33rd, 34th, 35th, 36th, 37th, 39th, 40th, 41st, 42nd, 43rd, 44th, 45th, 51st, 56th, 57th, and 58th items were noncompliant and were deleted, leaving a total of 28 items meeting the criteria. In addition, the three dimensions were analyzed for reliability and validity. The results showed that the alpha coefficients of the three dimensions and the alpha coefficients of the before and after parts of the scale were all higher than 0.90, indicating that the entries of the IHLS-CSMK scale have good consistency and homogeneity and are highly reliable.

Based on the above systematic and scientific quantitative analysis procedure, the official version was developed and contained 28 items. Structural equation modeling CFA was conducted on 412 participants using the IHLS-CSMK (official version). Three-factor structural model fit indices, *χ*^2^/df, GFI, AGFI, IFI, NFI, TLI, CFI, RMR, and RMSEA, all achieved a good fit to the model. This proves that the EFA and CFA analyses found the three-factor structure of interactive health literacy among college students majoring in kinesiology to be reasonable and effective. Therefore, the structure of IHLS-CSMK is reasonable and validated. Overall, this research culminates in a validated IHLS-CSMK model.

The IHLS-CSMK shows that there is an interrelated relationship between the dimensions of the structural model of interactive health literacy of physical education college students, and that each component exhibits different connotations of the group, which can directly affect interactive health literacy’s level of college kinesiology students. The dimension of mastering health information refers to the overall mastery of health literacy by college kinesiology students. The promoting healthy interactions dimension points to the application and dissemination of health information acquired by students of physical education. The solving health problems dimension indicates that physical education college students use the health information they have acquired to solve health problems, ultimately changing their own and others’ health status.

The IHLS-CSMK developed by our research institute has unique research value compared to existing health literacy scales. First, the IHLS-CSMKG assesses interactive health literacy, which can highlight the evaluation of interactive health literacy more than existing health literacy assessment scales. The IHLS-CSMK is an independent assessment scale specifically designed for interactive health literacy and is not an individual item included in the health literacy assessment scale. Second, the IHLS-CSMK targets the Chinese sports major college student population, which fills a research gap in this group. It can evaluate the interactive health literacy of this group, provide basic data for subsequent health education, and understand the actual health literacy level of this group, providing a scientific basis for relevant departments to formulate strategies to improve health literacy. Finally, the IHLS-CSMK developed by our research institute utilized a combination of qualitative and quantitative research methods. The author constructed a structural model of interactive health literacy among sports major college students through preliminary qualitative research, and then scientifically validated the developed structural model using quantitative research methods, demonstrating that it is a reasonable and reliable tool for evaluating interactive health literacy among sports major college students. This reflects the authors’ in-depth research in this field and the scientific application of research methods.

### 4.1 Conclusion

At present, research on health literacy in China is in its infancy, with a focus on disseminating health-related knowledge and using health education and knowledge to improve the overall health literacy of the population. The lack of comprehensive assessment tools has led to a low level of overall health literacy in China, and a lack of research on effective health communication channels and health education knowledge content. However, research teams in other countries have conducted more comprehensive studies on health literacy, allowing for deeper research from clinical medicine and public health perspectives. The assessment tools are relatively complete, and 32 scales have been developed to measure health literacy levels for different populations and levels of health literacy. Currently, there are no assessment scales developed to measure interactive health literacy and critical health literacy.

The health literacy level of college students can directly reflect the health literacy level of China’s high-quality population. College students with high health literacy can drive the high-quality development of the entire social health cause. Domestic and foreign researchers have found that the major studied by college students can affect their level of health literacy, and health education at the university level can promote the improvement of college students’ level of health literacy. Improving interactive health literacy mainly involves conducting health education in public places such as schools or communities, with sports as a direct means of promoting students’ health literacy. Therefore, from the perspective of public health, this study takes sports majors as the research object, explores the composition of their interactive health literacy, and develops an evaluation scale to expand the research boundary in the field of health literacy. At the same time, it improves the relevant research on interactive health literacy and deepens the theoretical and practical research on health literacy.

This study developed and validated the Interactive Health Literacy Assessment Scale for Sports Major College Students (IHLS-CSMKG), providing a scientific and effective tool for measuring and improving the health literacy level of this group. It can be used to identify current issues reflected in the health literacy of sports major college students and provide guidance for administrative departments to design and improve the health literacy level of sports major college students.

Based on the IHLS-CSMKG developed by our research institute, four suggestions are proposed to enhance the interactive health literacy of sports major college students.

(1) Optimize health education courses and training. First, enhance the interactivity of health education. The existing health education courses often focus on imparting knowledge, lacking interactivity and practicality. By introducing more case studies, situational simulations, and other teaching methods, the interactivity of the course can be enhanced, allowing students to solve health problems in simulated environments and improve their practical application abilities. Second, pay attention to the dissemination and communication skills of health information. By setting up specialized courses on health communication and health decision-making, students are trained on how to convey health knowledge to others and effectively communicate and persuade to enhance their ability to disseminate health information. Finally, provide practical opportunities to address health issues. By organizing health themed lectures, sports events, and other activities, students are encouraged to apply the health knowledge they have learned to practical life, enhancing their ability and confidence in solving health problems.

(2) Enhance digital and technological support. First, utilize intelligent technology and platforms. By developing digital platforms such as health management apps, online courses, and virtual laboratories, students can easily access health information, conduct health self-tests, and manage their health. These platforms can be combined with physical education courses to provide personalized health information recommendations and health behavior guidance. Second, enhance healthy interaction on social platforms. By utilizing social media and online communities, encourage students to share their experiences and challenges in healthy living, motivate more students to participate in health interactions, and promote the dissemination and sharing of health knowledge.

(3) Strengthen diversified health literacy assessment and feedback. By regularly using the IHLS-CSMK to assess the interactive health literacy of sports major college students, collecting data and providing personalized feedback. Based on the evaluation results, develop specific improvement plans and provide targeted training content according to the needs of different students. Interdisciplinary collaboration in sports, medicine, psychology, and sociology can provide comprehensive support for enhancing the interactive health literacy of sports majors. For example, psychology courses can help students better understand the psychological motivations behind healthy behaviors, while medical courses can provide scientific evidence for health knowledge.

(4) Support and motivation for campus environment. First, organize health promotion activities. Schools should regularly organize health education activities, such as health lectures, exercise and health weeks, to create a positive and healthy atmosphere and encourage students to participate. Second, establish a reward mechanism. For students who demonstrate outstanding performance in health literacy, schools can establish scholarships, certificates, and other forms of rewards to encourage more students to pay attention to and improve their own health literacy.

### 4.2 Limitations and future research

Due to the fact that no research is impeccable, there are some limitations to the development and validation of IHLS-CSMK. Given the limited sample size and location of this study, it may not represent all sports major university students worldwide, which could affect the generalizability of our research results. Therefore, it is recommended that future research include larger sample sizes and wider sample locations to further validate and improve the scale, enhance its applicability and scientific accuracy. Due to differences in cultural backgrounds, health concepts, and education systems, cross-cultural validation is still needed to determine whether this scale can be applied to other countries. To compensate for the shortcomings of this study, when implementing the IHLS-CSMK scale for cross-cultural validation in other countries, (1) the validation country should be clearly defined. Firstly, Asian countries that are closer to China can be selected for the survey, such as South Korea, Japan, India, etc., and then validation can be conducted in more countries. (2) Localized adjustment of the scale, including consideration of cultural differences such as language expression, differences in health concepts, differences in education systems, etc. Through collaboration with local health experts and educators, ensure the applicability of the scale locally. (3) Adopting stratified random sampling or cluster sampling to ensure sufficient sample size, and combining online and offline data collection methods to improve coverage of specific groups. (4) By combining quantitative and qualitative analysis methods, a balance between local and external perspectives can be achieved to scientifically and rationally analyze research results. This will provide a scientific, objective, and comprehensive understanding of the interactive health literacy of global sports majors, a special group related to health promotion, and provide reference for improving the health literacy level of college students and effectively promoting their professional development. This will help evaluate the validity and reliability of the scale in different cultural contexts, and provide data support for improving the health literacy of sports majors among college students worldwide.

In addition, to ensure that IHLS-CSMK is an effective and reliable assessment tool for measuring the level of interactive health literacy among sports majors, it is important to tailor the scale to meet the specific needs of those targeted. Given the different connotations of the three levels of health literacy (functional literacy, interactive literacy, and critical literacy), it is recommended that the IHLS-CSMK be revised to meet these needs. This may include changing the scale’s language, adjusting the format of the questions, or inclusion of health literacy levels that are relevant to the specific context of physical education. By doing so, the IHLS-CSMK could more accurately assess the overall level of health literacy in this population, which could inform the state’s development of effective health enhancement strategies that could contribute to the overall population’s health literacy level. For example, in some countries with low health literacy, it may be necessary to strengthen basic health education, whereas in countries with high health literacy, emphasis can be placed on improving interactive health literacy, especially in terms of health communication and problem-solving abilities.

## Data Availability

The datasets presented in this study can be found in online repositories. The names of the repository/repositories and accession number(s) can be found in the article/Supplementary material.
