# Supplementary material for: Development and validation of the interactive health literacy scale for college students majoring in kinesiology
Source: Front Psychol. 2026 Jan 6;16:1647504. doi: 10.3389/fpsyg.2025.1647504 (PMC12815786; doi:10.3389/fpsyg.2025.1647504)
Supplement: Supplementary file 1 [file Table_1.DOCX]

**Table 1 t-test for high and low grouping of each item in IHLS-CSMK (Pre-test version)（n=123）**

| **Items** | ***t*** | ***P*** |
| --- | --- | --- |
| 1.Ability to utilize new media communication tools. | 3.795 | 0.000*** |
| 2.Being in good health is more persuasive when communicating health messages. | 7.378 | 0.000*** |
| 3.Being able to get more health information from schools. | 8.816 | 0.000*** |
| 4.Physical health is the basic state of life. | 9.95 | 0.000*** |
| 5.Being able to get some health information from people around you. | 8.054 | 0.000*** |
| 6.Exercise requires specialized knowledge of health and exercise. | 10.164 | 0.000*** |
| 7.Better understanding of health information in the context of one's own experience. | 10.44 | 0.000*** |
| 8.Ability to search for many health-related information on the Internet. | 7.487 | 0.000*** |
| 9.Be able to identify true and valid health information. | 8.619 | 0.000*** |
| 10.Be able to acquire more health information through practical social experiences. | 12.189 | 0.000*** |
| 11.Be able to consult teachers or professionals to help them understand health information. | 8.879 | 0.000*** |
| 12.More health promotion can make college students pay more attention to health. | 11.125 | 0.000*** |
| 13.Have good communication skills to persuade others when disseminating health information. | 8.242 | 0.000*** |
| 14.Be able to ensure the accuracy of the health information disseminated. | 10.533 | 0.000*** |
| 15.More warning cases can arouse college students' attention to health. | 10.301 | 0.000*** |
| 16.The examination can strengthen the mastery of health information and improve health literacy. | 6.349 | 0.000*** |
| 17.To develop exercise habits in daily life according to their own situation. | 9.466 | 0.000*** |
| 18.Enhancing the knowledge of prevention of common diseases and medication can show health awareness. | 9.899 | 0.000*** |
| 19.Enhancement of health awareness through learning health courses. | 9.815 | 0.000*** |
| 20.Good social interaction skills can help to make correct health decisions. | 8.164 | 0.000*** |
| 21.Eating breakfast and not snacking is a healthy eating habit. | 3.331 | 0.001** |
| 22.Mental health problems can affect physical health. | 10.214 | 0.000*** |
| 23.Healthy living requires behavioral habits such as eating, resting and exercising. | 9.693 | 0.000*** |
| 24.Engaging in regular physical activity to maintain one's health status. | 9.043 | 0.000*** |
| 25.Staying healthy for self-esteem and others' perceptions. | 2.949 | 0.004* |
| 26.Schools can provide more health interventions to promote health awareness. | 12.943 | 0.000*** |
| 27.Health information can be directly disseminated in school-organized health promotion activities. | 9.266 | 0.000*** |
| 28.To alleviate physical ailments through exercise. | 10.58 | 0.000*** |
| 29.Long-term exercise habits enhance physical fitness and prevention of chronic diseases. | 11.165 | 0.000*** |
| 30.Healthy lifestyle habits and exercise behaviors require strong self-discipline. | 9.866 | 0.000*** |
| 31.Pre-exercise warm-up and post-exercise stretching are effective in preventing sports injuries. | 9.724 | 0.000*** |
| 32.Solving health problems requires knowledge of health status. | 8.164 | 0.000*** |
| 33.Lifestyle habits related to health care can affect physical health. | 7.212 | 0.000*** |
| 34.Being able to interpret health information correctly. | 11.083 | 0.000*** |
| 35.Being able to access health information from the WeChat public platform. | 9.604 | 0.000*** |
| 36.Need to have the means and channels of dissemination when sharing health information. | 11.59 | 0.000*** |
| 37.Be able to perform standard exercise movements that promote physical fitness. | 12.102 | 0.000*** |
| 38.Physical exercise can make the body in better shape. | 10.532 | 0.000*** |
| 39.The declining health status of college students can draw attention to the importance of health. | 8.735 | 0.000*** |
| 40.Different motivations for health can lead to different lifestyles. | 9.674 | 0.000*** |
| 41.Different health statuses for different physical qualities. | 7.531 | 0.000*** |
| 42.When encountering health problems, they will take the initiative to share and exchange health information with others. | 11.121 | 0.000*** |
| 43.Be able to acquire knowledge of health-promoting exercises. | 10.537 | 0.000*** |
| 44.Pay more attention to the health information related to themselves. | 10.365 | 0.000*** |
| 45.Exercise experience can help determine health problems such as sports injuries. | 12.002 | 0.000*** |
| 46.Physical activity enhances physical fitness and related motor skills. | 13.664 | 0.000*** |
| 47.Preventing sports injuries through good preparation for exercise. | 10.959 | 0.000*** |
| 48.Long-term healthy lifestyles and behaviors can increase health awareness. | 12.465 | 0.000*** |
| 49.Visualization of external devices enables monitoring of one's own well-being. | 11.348 | 0.000*** |
| 50.Regular participation in physical activities enhances social interaction skills. | 9.366 | 0.000*** |
| 51.Being able to socialize and have good interpersonal relationships is a sign of mental health. | 9.107 | 0.000*** |
| 52.People with health management needs will pay more attention to their diet. | 9.813 | 0.000*** |
| 53.Dynamic balance between energy intake and output can promote health. | 10.076 | 0.000*** |
| 54.Maintaining a regular work routine and not staying up late too often. | 11.114 | 0.000*** |
| 55.Regular physical activity can regulate mental health status. | 9.562 | 0.000*** |
| 56.A good state of health can address the psychological stress of training or studying. | 11.379 | 0.000*** |
| 57.Maintaining healthy habits can help make good health decisions. | 8.958 | 0.000*** |
| 58.Different groups of people participate in sports for higher pursuits such as body building. | 10.104 | 0.000*** |
| 59.Exercise experiences can help to better understand related chronic exercise injuries and diseases. | 10.893 | 0.000*** |
| 60.Health is more relevant to sport and can be more easily understood. | 9.46 | 0.000*** |

**TABLE S2 Information of participants for item analysis by IHLS-CSMK (Pretest version)（n=123）**

| **Variables** | **Content** | **Fre.** | **Percent(%)** |
| --- | --- | --- | --- |
| Sex | Male | 47 | 38.21 |
|  | Female | 76 | 61.79 |
| Ethnicity | Han | 89 | 72.36 |
|  | Minority | 34 | 27.64 |
| Grade | First | 20 | 16.26 |
|  | Second | 26 | 21.14 |
|  | Third | 37 | 30.08 |
|  | Fourth | 40 | 32.52 |
| Location | Eastern | 43 | 34.96 |
|  | Middle | 36 | 29.27 |
|  | Western | 23 | 18.70 |
|  | North-Eastern | 21 | 17.07 |
| Type of university | Comprehensive University | 44 | 35.77 |
|  | Normal College | 23 | 18.70 |
|  | Sports College | 56 | 45.53 |
| Level of university | 985 | 34 | 27.64 |
|  | 211 | 32 | 26.02 |
|  | Key | 36 | 29.27 |
|  | Average | 21 | 17.07 |

**Table S3 Information of participants for exploratory factor analysis (EFA)**

**by IHLS-CSMK (Beta version)（n=429）**

| **Variables** | **Content** | **Fre.** | **Percent(%)** |
| --- | --- | --- | --- |
| Sex | Male | 252 | 58.74 |
|  | Female | 177 | 41.26 |
| Ethnicity | Han | 296 | 68.99 |
|  | Minority | 133 | 31.01 |
| Grade | First | 139 | 32.41 |
|  | Second | 95 | 22.14 |
|  | Third | 106 | 24.71 |
|  | Fourth | 89 | 20.74 |
| Location | Eastern | 180 | 41.96 |
|  | Middle | 105 | 24.48 |
|  | Western | 53 | 12.35 |
|  | North-Eastern | 91 | 21.21 |
| Type of university | Comprehensive University | 147 | 34.27 |
|  | Normal College | 152 | 35.43 |
|  | Sports College | 130 | 30.30 |
| Level of university | 985 | 134 | 31.23 |
|  | 211 | 83 | 19.35 |
|  | Key | 60 | 13.99 |
|  | Average | 152 | 35.43 |

**TABLE S4 Information of participants for confirmatory factor analysis**

**(CFA) by IHLS-CSMK (Final version)（n=412）**

| **Variables** | **Content** | **Fre.** | **Percent(%)** |
| --- | --- | --- | --- |
| Sex | Male | 280 | 67.96 |
|  | Female | 132 | 32.04 |
| Ethnicity | Han | 244 | 59.22 |
|  | Minority | 168 | 40.78 |
| Grade | First | 118 | 28.64 |
|  | Second | 63 | 15.29 |
|  | Third | 126 | 30.58 |
|  | Fourth | 105 | 25.49 |
| Location | Eastern | 170 | 41.26 |
|  | Middle | 127 | 30.82 |
|  | Western | 55 | 13.36 |
|  | North-Eastern | 60 | 14.56 |
| Type of university | Comprehensive University | 131 | 31.80 |
|  | Normal College | 129 | 31.31 |
|  | Sports College | 152 | 36.89 |
| Level of university | 985 | 109 | 26.46 |
|  | 211 | 96 | 23.30 |
|  | Key | 133 | 32.28 |
|  | Average | 74 | 17.96 |

**TABLE S5 Correlation between the 60 items and total score of IHLS-CSMK (Pre-test version)（n=123）**

| **Number** | **Average** | **Standard deviation** | **Correlation coefficient** | **Number** | **Average** | **Standard deviation** | **Correlation coefficient** |
| --- | --- | --- | --- | --- | --- | --- | --- |
| **1** | 4.2 | 0.884 | **.453**** | **31** | 4.4 | 0.637 | **.789**** |
| **2** | 4.37 | 0.76 | **.638**** | **32** | 4.33 | 0.683 | **.788**** |
| **3** | 4.07 | 0.776 | **.652**** | **33** | 4.21 | 0.727 | **.703**** |
| **4** | 4.3 | 0.689 | **.729**** | **34** | 4.06 | 0.761 | **.762**** |
| **5** | 4.06 | 0.739 | **.680**** | **35** | 3.96 | 0.863 | **.724**** |
| **6** | 4.34 | 0.687 | **.689**** | **36** | 4.1 | 0.706 | **.795**** |
| **7** | 4.27 | 0.654 | **.766**** | **37** | 4.11 | 0.733 | **.812**** |
| **8** | 4.2 | 0.735 | **.657**** | **38** | 4.34 | 0.638 | **.784**** |
| **9** | 3.95 | 0.777 | **.680**** | **39** | 4.19 | 0.705 | **.710**** |
| **10** | 4.16 | 0.658 | **.786**** | **40** | 4.27 | 0.678 | **.786**** |
| **11** | 4 | 0.84 | **.656**** | **41** | 4.28 | 0.659 | **.734**** |
| **12** | 4.21 | 0.681 | **.776**** | **42** | 4.1 | 0.804 | **.742**** |
| **13** | 4.25 | 0.673 | **.688**** | **43** | 4.07 | 0.77 | **.763**** |
| **14** | 4.07 | 0.797 | **.703**** | **44** | 4.23 | 0.699 | **.768**** |
| **15** | 4.23 | 0.584 | **.755**** | **45** | 4.2 | 0.786 | **.802**** |
| **16** | 3.93 | 0.847 | **.558**** | **46** | 4.28 | 0.669 | **.804**** |
| **17** | 4.15 | 0.698 | **.729**** | **47** | 4.26 | 0.663 | **.809**** |
| **18** | 4.23 | 0.612 | **.778**** | **48** | 4.32 | 0.618 | **.799**** |
| **19** | 4.22 | 0.672 | **.721**** | **49** | 4.19 | 0.657 | **.786**** |
| **20** | 4.17 | 0.71 | **.705**** | **50** | 4.15 | 0.743 | **.725**** |
| **21** | 4.23 | 0.847 | **.384**** | **51** | 4.2 | 0.757 | **.749**** |
| **22** | 4.46 | 0.618 | **.745**** | **52** | 4.25 | 0.731 | **.782**** |
| **23** | 4.41 | 0.664 | **.740**** | **53** | 4.26 | 0.598 | **.796**** |
| **24** | 4.2 | 0.816 | **.689**** | **54** | 4.28 | 0.693 | **.735**** |
| **25** | 3.42 | 1.145 | **.324**** | **55** | 4.37 | 0.658 | **.785**** |
| **26** | 4.1 | 0.773 | **.753**** | **56** | 4.36 | 0.616 | **.787**** |
| **27** | 4.08 | 0.685 | **.752**** | **57** | 4.28 | 0.657 | **.790**** |
| **28** | 4.23 | 0.675 | **.763**** | **58** | 4.17 | 0.721 | **.763**** |
| **29** | 4.32 | 0.644 | **.814**** | **59** | 4.15 | 0.786 | **.776**** |
| **30** | 4.33 | 0.671 | **.812**** | **60** | 4.24 | 0.669 | **.740**** |

**TABLE S6 Results of the correlation matrix for the IHLS-CSMK (Beta version)（n=429）**

|  | Harnessing Health Information | Promoting Health Interactions | Addressing Health Problems | Total scale |
| --- | --- | --- | --- | --- |
| Harnessing Health Information | 1.000 | .577** | .593** | .715** |
| Promoting Health Interactions | .577** | 1.000 | .847** | .955** |
| Addressing Health Problems | .593** | .847** | 1.000 | .947** |
| Total scale | .715** | .955** | .947** | 1.000 |

**TABLE S7 Three factors and alpha coefficients of IHLS-CSMK（n=429）**

| **Factors** | **Harnessing Health Information** | **Promoting Health Interactions** | **Addressing Health Problems** | **IHLS-CSMK** |
| --- | --- | --- | --- | --- |
| Internal consistency  reliability (α coefficient) | 0.794 | 0.943 | 0.932 | 0.962 |
